# A clinicopathologic analysis of microscopic extension in small cell lung cancer and lung adenocarcinoma: Determination of clinical target volume with precise radiotherapy

**DOI:** 10.1111/1759-7714.14000

**Published:** 2021-05-24

**Authors:** Liwei Gao, Xiuhong Wang, Xiongtao Yang, Runchuan Gu, Guangying Zhu, Xianshu Gao

**Affiliations:** ^1^ Department of Radiation Oncology Peking University First Hospital Beijing China; ^2^ Department of Pathology China‐Japan Friendship Hospital Beijing China; ^3^ Department of Radiation Oncology China‐Japan Friendship Hospital Beijing China

**Keywords:** clinical target volume, intensity‐modulated radiation therapy, lung cancer, microscopical extension

## Abstract

**Purpose:**

The identification of the clinical target volume (CTV) is particularly important in the precise radiotherapy of lung cancer. The purpose of this study was to determine the extension margin from gross tumor volume (GTV) to CTV in primary small cell lung cancer (SCLC) and lung adenocarcinoma (ADC) by microscopic extension (ME).

**Material and Methods:**

The data of 25 cases of SCLC and 29 cases of ADC from August 2015 to August 2020 were analyzed. The measurement of tumor size between preoperative thoracic computed tomography (CT) and postoperative macroscopic specimens was compared, and the ME range of tumor cells was measured under a microscope to determine its correlation with clinical features and pathological manifestations.

**Results:**

A total of 217 slides were examined, corresponding to 103 slides for SCLC and 114 slides for ADC. The radiologic sizes of the tumors in SCLC and ADC were 12.8 and 7.9 mm, respectively (*p* = 0.09), and the macroscopic sizes were 12.5 and 8.5 mm, respectively (*p* = 0.07). There was a significant correlation between the radiologic and macroscopic size of the same tumor sample (r = 0.886). Compared with ADC, more SCLC tumor cells infiltrated through vascular or lymphatic dissemination (16% vs. 9%, *p* = 0.047). The mean ME value was 2.81 mm for SCLC and 2.02 mm for ADC (*p* = 0.012). To take into account 95% of the ME, a margin of 8 and 7.7 mm must be expanded for SCLC and ADC, respectively. The ME value of the tumor was related to the presence of atelectasis, the location of the tumor, and the Ki‐67 cell proliferation index.

**Conclusion:**

The GTV of the tumor was contoured according to CT images, which was basically consistent with the actual tumor size. The GTVs of SCLC and ADC should be expanded by 8 and 7.7 mm, respectively, to fully cover the subclinical lesions in 95% of cases.

## Graphical abstract

The purpose of this study is to optimize the delineation range of the CTV in precise radiotherapy to increase the radiation dose of the tumor and reduce the radiation dose of normal tissue at the same time, the GTVs of SCLC and ADC should be expanded by 8 and 7.7 mm, respectively, to fully cover the subclinical lesions in 95% of cases.

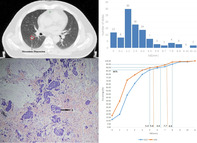

## INTRODUCTION

Worldwide, lung cancer ranks first in morbidity and mortality^1^ of malignancies and can be classified into non‐small cell lung cancer (NSCLC) and small cell lung cancer (SCLC). NSCLC mainly includes squamous cell carcinoma (SCC) and adenocarcinoma (ADC). Radiotherapy plays an important role in the treatment of both NSCLC and SCLC.^2^, ^3^, ^4^ The basic principles of radiation therapy for lung cancer are as follows: (1) use a high‐dose radiation range that includes all of the macroscopic tumor volume and microscopic extension, and (2) reduce the radiation dose of surrounding lung tissue and normal organs as much as possible.^5^ In recent years, three‐dimensional conformal radiation therapy (3D‐CRT) and intensity‐modulated radiation therapy (IMRT), as representative precise radiotherapy technologies, have provided the possibility of increasing the radiation dose of tumors and reducing normal tissue damage. In 1993, the International Commission on Radiation Units and Measurements (ICRU) published Report No. 50,^6^ which mainly includes the contents and suggestions for gross tumor volume (GTV), clinical target volume (CTV), and planning target volume (PTV) in the target area of external photon beam irradiation. IRCU Report No. 62^7^ published in 1999 as a supplement to ICRU 50, distinguishes some concepts and definitions of tumor motion and setup margin. However, the original contents of GTV and CTV have not been modified. GTV includes tumor volume displayed by palpation or imaging, and CTV includes GTV and microscopic extension (ME) range (subclinical lesions) around the tumor, but the scope of CTV is not specified. With the development of computed tomography (CT), magnetic resonance imaging (MRI), positron emission tomography (PET)‐CT, and other imaging technologies, the display of thoracic tumors is becoming increasingly accurate^8^, ^9^, ^10^; however, no imaging method can accurately display the subclinical lesions around the tumor. The determination of the ME range of tumor cells is very complicated. It may be related to the histological type, anatomical location, size, differentiation, and various clinical or pathological factors of the tumor.^11^ Moreover, in the three‐dimensional direction, the scope of ME is also inconsistent. It is very difficult and important for radiotherapists to define the limit of CTV because the key to the implementation of precise radiotherapy lies in the determination of the tumor target volume and surrounding subclinical lesions, which is directly related to the final efficacy and toxicity of radiotherapy. The purpose of this study was to determine the ME value and its correlation with the clinicopathologic characteristics of patients with SCLC and ADC after lobectomy or wedge resection and to define the CTV of SCLC and ADC as precisely as possible.

## MATERIALS AND METHODS

### Patients

From October 2016 to October 2020, 25 SCLC and 29 ADC specimens treated by lobectomy or wedge resection in China‐Japan Friendship Hospital were included. We fixed the specimens with 10% formalin solution, and tumor and adjacent lung tissues were collected. Among the histological slides examined, those for which we could clearly identify the tumor margin and its relationship with normal lung or bronchial parenchyma were selected for study. There were 103 SCLC slides and 114 ADC slides in total (see Table 1).

**TABLE 1:** Patient characteristics

	Small cell lung cancer		Adenocarcinomas		Total		*p*
	*n*	(%)	*n*	(%)	*n*	(%)	
Patients	25	(46)	29	(54)	54	100	NS
Slides^a^	103	(47)	114	(53)	217	100	NS
Age(mean, in years)	60.1		61.3		60.7		0.004
Gender							
Male	19	(76)	11	(38)	30	(56)	0.005
Female	6	(24)	18	(62)	24	(44)	
Stage							
I	14	(56)	23	(79)	37	69	NS
II	7	(28)	4	(14)	11	20	NS
III	4	(16)	2	(7)	6	11	NS
IV	0	(0)	0	(0)	0	0	NS
Atelectasis							
Yes	3	(12)	0	(0)	3	6	NS
No	22	(88)	29	(100)	51	94	
Site							
Proximal	11	(44)	0	(0)	11	20	<0.001
Peripheral	14	(56)	29	(100)	43	80	
Mode of extension^a^							
Vascular	15	(16)	8	(9)	23	12	0.047
Local	76	(84)	86	(91)	162	88	
Adjacent lung^a^							
Normal	89	(86)	114	(100)	203	94	<0.001
Pathologic	14	(14)	0	(0)	14	6	
Ki‐67 index(mean)		79.1		23.2		51.5	<0.001

Abbreviation: NS, no significance.

^a^ Number of slides presenting this criterion.

### Histological classification

The histological classification of each case selected was based on the latest World Health Organization (WHO) 2015 classification standard.^12^ The macroscopic dimension of the tumor, lymph node metastasis status, presence of lymphatic or blood vessel invasion, and Ki‐67 index were determined and recorded by professional pathologists according to the classic morphological criteria of lung cancer.^13^, ^14^ Based on the relationship between the tumor site and lobar or segmental bronchi, the tumor was defined as proximal or peripheral lung cancer. Proximal lung cancers were defined as tumors situated by the hilum of the lung and arising from the main bronchus, lobar bronchi, or segmental bronchi. Peripheral lung cancer was defined as that arising below segmental bronchi and located in the periphery of the lung.^15^

### Validity of technology

In the study of tumor ME, we first ensured that there were sufficient normal lung tissues around the tumor in each slide that the ME range of tumor cells could be fully measured. The margin of the tumor was identified with the naked eye and outlined with a marker pen. The value of the local ME outside of this border was measured under a ×5 times (suspicious case ×10 times) light microscope with an eyepiece micrometer.

**FIGURE 1:**
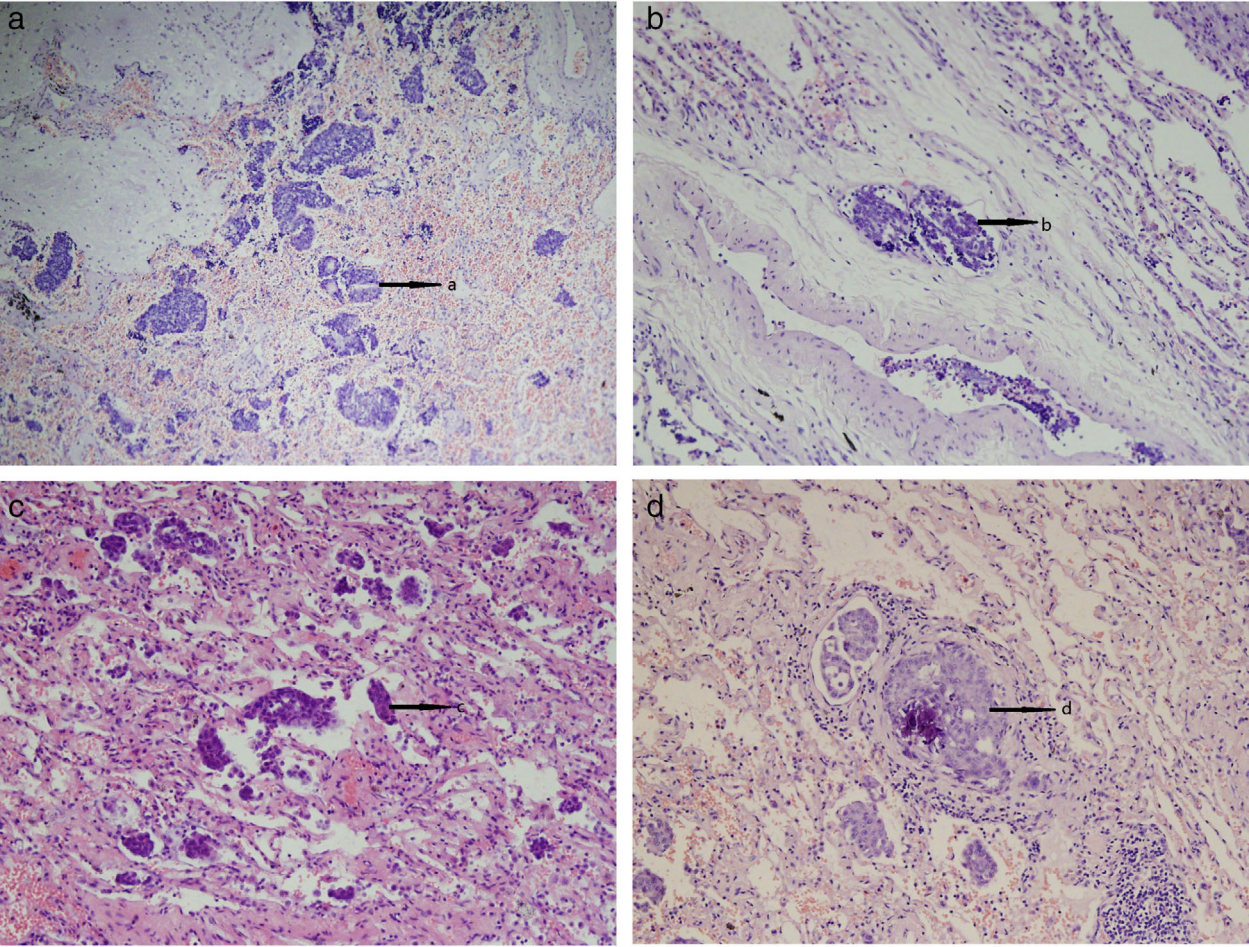
(a) Aerogenous microscopic extension in a SCLC (a: metastatic cells in an alveolus, ×20). (b) Hematogenous microscopic extension in a SCLC (b: metastatic extension in a blood vessel, ×20). (c) Aerogenous microscopic extension in an adenocarcinoma (c: metastatic cells in an alveolus, ×20). (d) Hematogenous microscopic extension in an adenocarcinoma (d: metastatic extension in a blood vessel, ×20)

According to the terms described and revised by Spencer^13^ and Colby,^14^ the patterns of tumor extension were evaluated, including (1) direct alveolar extension (i.e., spreading along the alveolar wall or pre‐existing structures) with no modification of the basement membrane (i.e., with the presence of tumor cells in the interstitium pulmonary), (2) aerogenic dissemination, defined as the presence of free tumor cells in the alveolar cavity, and (3) vascular or lymphatic dissemination. The (1) and (2) patterns were defined as “local extension” (as shown in Figure 1(a),(b)), and the (3) pattern was defined as “vascular extension” (Figure 1(c),(d)). In addition, the lung parenchyma around the tumor was classified as “normal” or “pathological” according to the state of the lung parenchyma around the tumor, and the “pathological” state included bleeding, fibrosis, and intraluminal and/or interstitial inflammation.

To ensure the accuracy of operation, all patients underwent breath‐holding chest high‐resolution CT examination 1–3 days before operation. The scanning parameters used were as follows: tube voltage, 120 kV; tube current‐exposure time product, 150 mAs; rotation time, 0.5 s; pitch, 0.6; and slice thickness, 1.0 mm, lung window settings (window width, 1200 HU; window level, −600 HU), and mediastinal window settings (window width, 250 HU; window level, 40 HU).

**FIGURE 2:**
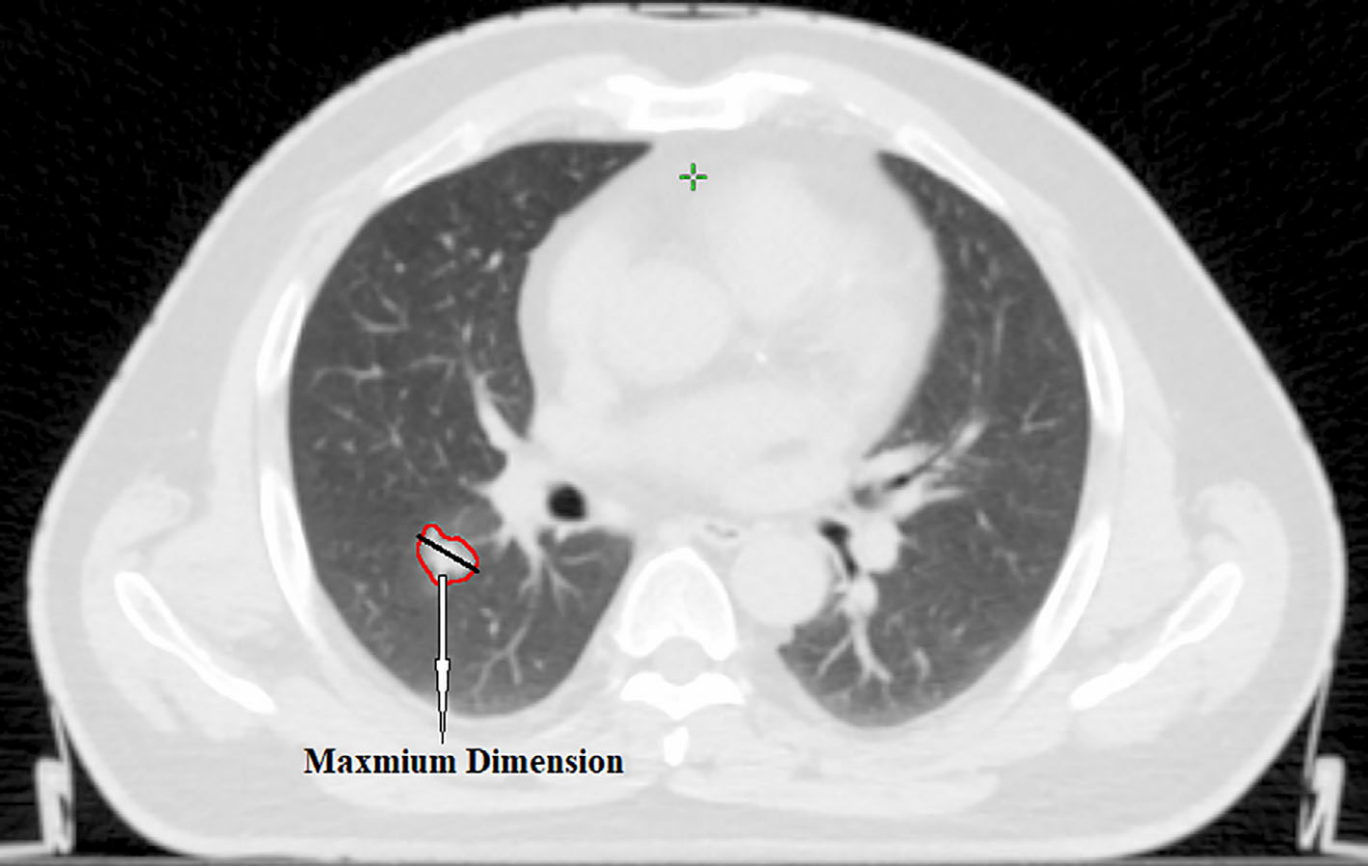
Maximal tumor size as measured on computed tomography (CT) lung windows

According to the clinical and radiologic data of the patients, the following parameters were collected: anatomic location of lung tumor, TNM and pTNM staging, and whether the presence of atelectasis or not. The radiologic size of tumor was measured in the condition of lung window by preoperative CT (see Figure 2).

### Statistical analysis

Statistical analysis was performed using the SPSS 23.0 statistical package. A paired *t*‐test and correlation coefficient analysis were used to analyze the correlation between radiologic and macroscopic tumor size. An independent sample *t*‐test was used to analyze the correlation between the various clinical or pathological features and ME. A χ2 test or Fisher's exact test was used to compare qualitative parameters.

## RESULTS

The clinical and pathological features of the patients are shown in Table 1. In this study, 103 pathological slides of 25 SCLC and 114 pathological slides of 29 ADC were analyzed. Patients with SCLC were more often men (76% vs. 68%, *p* = 0.005), and the mean age was significantly lower than that of patients with ADC (60.1 vs. 61.3, *p* = 0.004). TNM and pTNM staging were similar in two groups. The proportion of proximal lung cancer in the SCLC group was significantly higher than that in the ADC group (44% vs. 0%, *p* < 0.001). There were three cases of atelectasis in the SCLC group, but there was no significant difference between the two groups. According to the classification, the proportion of lung parenchyma surrounding the tumor considered “pathological” for SCLC was much higher than that for ADC: 14% and 0% (*p* < 0.001), respectively, and the major component was inflammatory. Compared with vascular dissemination, local extension was the main pattern of ME, and air source dissemination was the most common “local” extension mode in the two groups. The proportion of “local” extension was 84% in SCLC and 91% in ADC. Although the major ME mode in both groups was “local” extension, the proportion of “vascular” extension was significantly higher in SCLC than in ADC (16% vs. 9%, *p* = 0.047). The higher rate of “vascular” extension in SCLC may be associated with its higher inclination for lymph node and distant organ metastasis. The Ki‐67 index of SCLC was 79.1%, which was much higher than that of ADC (23.2%, *p* < 0.001), and it also showed that SCLC tumor cells had higher proliferation activity.

### Radio‐macroscopic correlations

**FIGURE 3:**
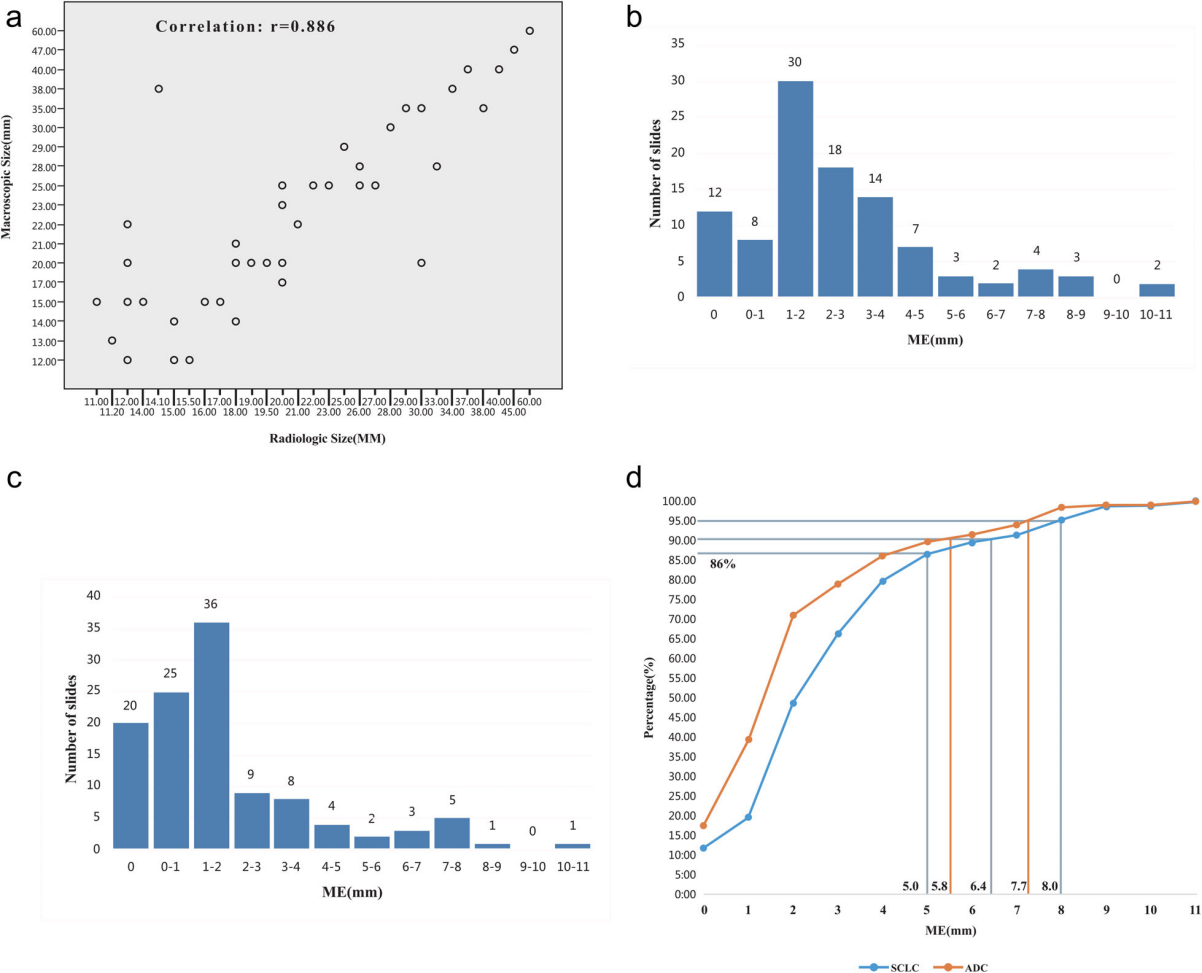
(a) Correlation between radiologic and macroscopic size. (b) Microscopic extension (ME) distribution in the SCLC group. (c) Microscopic extension (ME) distribution in the ADC group. (d) Cumulative distribution of microscopic extension (ME) in the SCLC and ADC groups

Without considering the ME value, the radiologic size of the tumor was slightly smaller than the macroscopic size postoperatively. The mean radiologic size of SCLC was 26.3 ± 12.8 mm (range:12–60 mm), and 20.8 ± 7.9 mm (range:11–40 mm) for ADC. SCLC was larger than ADC, but the difference was not statistically significant (*p* = 0.09). This difference was also observed on macroscopic measurement; SCLC was larger than ADC (28.4 ± 12.5 mm vs. 22.2 ± 8.5 mm, respectively, (*p* = 0.07), Table 2). However, the most important finding was that comparative analysis of the two measurements revealed a very significant correlation (r = 0.886, Figure 3(a)) between radiologic size and macroscopic size; therefore, it was reasonable and accurate to delineate the GTV of the primary tumor in the lung window condition of the CT image.

**TABLE 2:** Measurement of radiologic and macroscopic dimensions for the same patient

	*n*	Mean (mm)	SD (mm)	Range (mm)	*p*
Radiologic size					
Small cell lung cancer	25	26.3	12.8	12–60	0.09
Adenocarcinoma	29	20.8	7.9	11–40	
Macroscopic size					
Small cell lung cancer	25	28.4	12.5	0–10.5	0.07
Adenocarcinoma	29	22.2	8.5	0–10.1	

Abbreviation: SD, standard deviation.

We observed a significant difference between SCLC and ADC of ME (Table 3). The mean ME value was 2.81 ± 2.39 mm (range: 0–10.5 mm) for SCLC in 103 slides and 2.02 ± 2.18 mm (range:0–10.1) for ADC in 114 slides，(*p* = 0.012). These values are much smaller than the values for the healthy tissue margins adjacent to the tumor. Therefore, these measurement values can represent the maximum extension of tumor cells, and they are not related to the sample collection or measurement conditions. ME differences between the two histological groups existed, and distribution analysis also confirmed these differences (Table 4 and Table 5). Figure 3(b) and (c) present ME frequency tables classified by increments of 1 mm together, and Figure 3(d) presents the ME cumulative frequency table. We observed that to take into account 90% and 95% of the ME, GTV should be expanded by 6.4 and 8 mm to CTV in SCLC. To take into account 90% and 95% of the ME, GTV should be expanded by 5.8 and 7.7 mm in ADC.

**TABLE 3:** Distance of ME in mm

All slides	n	No. of slides	Mean (mm)	SD (mm)	Range (mm)	*p*
Small cell lung cancer	25	103	2.81	2.39	0–10.5	0.012
Adenocarcinoma	29	114	2.02	2.18	0–10.1	

Abbreviation: ME, microscopic extension; SD, standard deviation.

**TABLE 4:** ME distribution for SCLC

ME(mm)	No.	Cumulative	No. %	Cumulative %
0.00	12	12	11.65	11.65
0.05	1	13	0.97	12.62
0.50	1	14	0.97	13.59
0.70	2	16	1.94	15.53
0.80	1	17	0.97	16.50
0.90	1	18	0.97	17.48
1.00	2	20	1.94	19.42
1.10	2	22	1.94	21.36
1.20	5	27	4.85	26.21
1.30	2	29	1.94	28.16
1.40	2	31	1.94	30.10
1.50	3	34	2.91	33.01
1.60	1	35	0.97	33.98
1.70	2	37	1.94	35.92
1.80	3	40	2.91	38.83
1.90	4	44	3.88	42.72
2.00	6	50	5.83	48.54
2.10	2	52	1.94	50.49
2.20	2	54	1.94	52.43
2.30	1	55	0.97	53.40
2.50	3	58	2.91	56.31
2.60	2	60	1.94	58.25
2.70	1	61	0.97	59.22
2.80	2	63	1.94	61.17
2.90	2	65	1.94	63.11
3.00	3	68	2.91	66.02
3.10	4	72	3.88	69.90
3.20	2	74	1.94	71.84
3.50	4	78	3.88	75.73
3.70	1	79	0.97	76.70
3.80	1	80	0.97	77.67
3.90	1	81	0.97	78.64
4.00	1	82	0.97	79.61
4.40	1	83	0.97	80.58
4.50	2	85	1.94	82.52
4.60	2	87	1.94	84.47
4.80	1	88	0.97	85.44
5.00	1	89	0.97	86.41
5.30	1	90	0.97	87.38
5.50	1	91	0.97	88.35
6.00	1	92	0.97	89.32
6.40	1	93	0.97	90.29
6.50	1	94	0.97	91.26
7.20	1	95	0.97	92.23
7.60	1	96	0.97	93.20
8.00	2	98	1.94	95.15
8.60	1	99	0.97	96.12
8.90	2	101	1.94	98.06
10.50	2	103	1.94	100.00

Abbreviations: ME, microscopic extension; SCLC, small cell lung cancer.

**TABLE 5:** ME distribution for ADC

ME (mm)	No.	Cumulative	No. %	Cumulative %
0.00	20	20	17.54	17.54
0.10	1	21	0.88	18.42
0.40	2	23	1.75	20.18
0.50	1	24	0.88	21.05
0.60	7	31	6.14	27.19
0.70	4	35	3.51	30.70
0.80	1	36	0.88	31.58
0.90	4	40	3.51	35.09
1.00	5	45	4.39	39.47
1.10	5	50	4.39	43.86
1.20	3	53	2.63	46.49
1.30	2	55	1.75	48.25
1.40	2	57	1.75	50.00
1.50	6	63	5.26	55.26
1.60	4	67	3.51	58.77
1.80	2	69	1.75	60.53
1.90	2	71	1.75	62.28
2.00	10	81	8.77	71.05
2.10	2	83	1.75	72.81
2.20	3	86	2.63	75.44
2.30	1	87	0.88	76.32
2.40	1	88	0.88	77.19
2.70	1	89	0.88	78.07
3.00	1	90	0.88	78.95
3.20	1	91	0.88	79.82
3.50	1	92	0.88	80.70
3.60	3	95	2.63	83.33
3.80	2	97	1.75	85.09
4.00	1	98	0.88	85.96
4.20	1	99	0.88	86.84
4.30	3	102	2.63	89.47
5.00	1	103	0.88	90.35
6.00	1	104	0.88	91.23
6.10	1	105	0.88	92.11
6.20	1	106	0.88	92.98
7.00	1	107	0.88	93.86
7.10	1	108	0.88	94.74
7.70	1	109	0.88	95.61
7.80	2	111	1.75	97.37
8.00	1	112	0.88	98.25
9.00	1	113	0.88	99.12
10.10	1	114	0.88	100.00

Abbreviations: ME, microscopic extension; ADC, adenocarcinoma.

### Clinicopathological factors of ME

We also analyzed the correlation between the clinicopathologic characteristics and ME in the two groups. For SCLC, the presence of atelectasis was associated with a lower ME, and the mean ME value in patients with atelectasis was 1.55 mm, which was 2.97 mm for patients without atelectasis. There was a significant difference between the two groups (*p* = 0.049). In addition, the ME value of proximal SCLC was 3.2 mm, and that of peripheral SCLC was 2.32 mm. There was a trend of higher ME in proximal SCLC, but the difference was not statistically significant (*p* = 0.064). In terms of the Ki‐67 index, the mean values were taken as the cut off point; for ADC, the mean ME value was 2.42 mm for high Ki‐67 cases and 1.29 mm for low Ki‐67 cases (*p* = 0.011). In SCLC, the ME values of high and low Ki‐67 cases were 2.96 and 2.49 mm, respectively, and there was no significant difference between the two groups (*p* = 0.343).

There was no significant correlation between ME and patient age, sex, T stage, N stage, infiltration mode, or adjacent lung parenchyma. In addition, some samples did not show any ME, but we did not detect any characteristics of these samples.

## DISCUSSION

Because of the existence of ME around the tumor, the GTV delineated by CT images cannot completely cover the subclinical lesions around the primary tumor. Therefore, it is necessary to extend a certain range around the GTV to fully irradiate the primary tumor, which is particularly important in the current era of precise radiotherapy. In the comparative study of imaging and pathology of NSCLC, it was found that the ME of different pathological types was different. Giraud et al.^11^ found that the average ME value was 2.76 mm for lung ADC and 2.37 mm for SCC; if 95% of the scope of ME was covered, GTV extended to CTV, which required 8 mm for ADC and 6 mm for SCC. Grills et al.^16^ researched the ME of ADC with different nuclear grades. For a GTV contoured on the CT lung windows, the margin required to cover ME for 90% of the cases would be 9 mm (9, 7, and 4 mm for grades 1 to 3, respectively). Li et al.^17^ found that in the expanded region from GTV to CTV, ADC needs 7 mm, including 95% of the ME, whereas squamous cell carcinoma only needs 5 mm. Therefore, in the current clinical application, the GTV to CTV of ADC and SCC are 7–9 mm and 5–6 mm, respectively. However, because of the lack of standardized imaging and pathological control studies, there is no uniform standard for the formulation of CTV in SCLC, and each radiotherapy center varies greatly. In the RTOG9712 study, which was mainly based on conventional radiotherapy, the GTV was extended 1–1.5 cm to the CTV.^18^ In the concurrent once daily versus twice daily radiotherapy for limited stage small cell lung cancer (CONVERT)^19^ study, which was based on 3D‐CRT or IMRT, the GTV was extended by 0.5 cm to the CTV. This extension method was also recommended by the ESTRO Advisory Committee in Radiation Oncology (ACROP) guidelines.^20^

Our study reviewed the clinical and pathological data of 25 patients with SCLC and 29 patients with ADC from 2015 to 2020. First, it was determined that the radiologic size of the tumor measured on CT was closely related to the tumor size of macroscopic specimens without considering ME, and the imaging findings were consistent with the actual size of the tumor. This result provides a theoretical basis for the delineation of GTV. In terms of ME of tumors, more SCLC samples extended along the vessels than ADC, which may be related to the higher probability of lymphatic and hematogenous dissemination of SCLC. In this study, ADC was selected as the research object while targeting the ME of SCLC. The purpose was to verify the previous ADC research results and to provide quality control for the results of this study at the same time. The mean ME value of ADC is 2.02 mm. To cover 95% of the ME, ADC needs to be extended by 7.7 mm to CTV, which is basically consistent with previous clinical studies.^11^, ^16^, ^17^ The mean ME value of SCLC was 2.81 mm. To cover 95% of ME, SCLC needs to be expanded by 8 mm to CTV, which may be related to the stronger proliferation activity and greater invasiveness of SCLC tumor cells. The classical 5‐mm margin can only take into account 86% of the ME range.

We also studied the correlation between the ME values and the clinical, radiologic, and pathological characteristics of patients. These characteristics are the basic data that must be obtained before radiotherapy starts. The presence of atelectasis in SCLC limited the ME of the tumor, which may be because of the retraction of adjacent lung parenchyma restricting the ventilation of lung tissue around the tumor, which hindered the migration of tumor cells. However, the ME of central lung cancer tended to increase, but the difference was not statistically significant. The increase in Ki‐67 was positively correlated with the increase in ME, especially in the ADC group, and the difference was not found in the SCLC group, which may be related to the generally high Ki‐67 index. In contrast, ME does not seem to depend on macroscopic or microscopic tumor size or tumor stage. Finally, lymphatic and vascular dissemination are the main methods of distant metastasis of tumor cells,^21^ which is associated with poor prognosis of patients.^22^, ^23^ In our study, SCLC had a more extensive pattern of vascular invasion, which may have caused tumor cells to migrate rapidly to the peripheral region of the primary tumor; this is also associated with a higher metastatic rate and poorer prognosis of SCLC.^24^, ^25^

Conventional radiotherapy and 3D‐CRT cannot form a dose gradient between GTV, CTV, and PTV, but the development of IMRT makes the treatment of tumors no longer a uniform dose in the treatment field but a targeted dose between different areas^26^; therefore, it is more important to determine the scope of ME for tumors.

In principle, surgery is only applicable to SCLC patients with T1 or T2 stage disease and without lymph nodes or distant metastasis, so most of the patients enrolled in this study were early stage and small lesions, for these patients, if they are not suitable for or refuse surgical treatment, the clinical preferred treatment method was stereotactic body radiation therapy (SBRT^).^ ^27^, ^28^ The characteristics of SBRT, such as high precision, high dose and low fractions, make it possible to achieve a certain range of radiation agents outside PTV, Jin et al.^29^ found that the prescription dose of 64% ± 7% could still be achieved at 1 cm around the PTV with four‐dimensional (4D) CT localization, at the same time for reducing the side effects of SBRT, PTV is usually expanded directly outside internal gross target volume (IGTV) in clinical practice, which also shows a high local control rate.^30^, ^31^ The results of our study showed that PTV can be directly expanded outside IGTV for SBRT, it was enough to cover subclinical lesions in 8 mm for SCLC and 7.7 mm for ADC. However, for traditional fractionated radiotherapy, it is necessary to extend GTV to CTV according to different pathological types.

At present, programmed death‐1 (PD‐1)/programmed death ligand‐1 (PD‐L1) inhibitors play a very important role in the standard treatment of advanced NSCLC and extensive stage SCLC. The tumor cells and/or immunological cells in tumor tissues inhibit the antitumor immune response by expressing PD‐L1, whereas PD‐1 or PD‐L1 inhibitors can reactivate the inhibited T cells to kill tumor cells. The expression of PD‐L1 in NSCLC, renal cell carcinoma, melanoma, bladder cancer, and other types of solid tumors was related to the better efficacy of PD‐L1 inhibitors.^32^ It is not clear whether the expression of PD‐L1has prognostic value, but some studies^33^, ^34^ have shown that the expression of PD‐L1 was related to the response of stromal cells in tumor microenvironment. The immunosuppressive cytokines or proteases secreted by tumor associated immune cells can induce PD‐L1 overexpression, activate related signaling pathways, and increase invasiveness of the tumor cells, which may affect the ME of lung cancer cells. Because all of the specimens in our study were resectable lung cancer cases, stage of the tumors was relatively early, immunotherapy was not standard treatment method for the clinical application of these patients, PD‐L1 expression status has not been detected in these cases. In the further research, we will focus on exploring the relevance between some molecular biological markers (such as PD‐L1 expression) and the ME range of tumor cells.

Because of the limitations of surgery in the application of SCLC, the number of patients enrolled in this study is relatively small, and the data need to be verified by larger clinical studies.

## CONCLUSION

This was the first study focused on the invasive range of SCLC in the world; we first determined that it was reasonable to contour GTV according to CT images under appropriate lung windows. Second, we measured the ME range of SCLC and ADC under microscopic conditions. There was a significant difference in the mean ME value between the two groups; the mean ME value of SCLC was 2.81 mm and that of ADC was 2.02 mm. In the actual radiotherapy environment, if we want to cover 95% of the cases of subclinical lesions (i.e., with an error risk of 5%) the expansion range of GTV in SCLC and ADC is 8 and 7.7 mm, respectively, and the location of the tumor, presence of atelectasis and proliferation activity of tumor cells should be considered. The purpose of this study is to optimize the delineation range of the CTV in precise radiotherapy to increase the radiation dose of the tumor and reduce the radiation dose of normal tissue at the same time, which is also the primary goal of precise radiotherapy.

## CONFLICT OF INTEREST

We declare that we do not have any commercial or associative interest that represents a conflict of interest in connection with the work submitted.
